# Multiple branch retinal artery occlusions following the new facial cosmetic filler (Poly-D, L-lactic Acid) injection a case report

**DOI:** 10.1186/s12886-023-02821-8

**Published:** 2023-03-06

**Authors:** I Wang, Hui-Ju Lin, Yi-Yu Tsai, Wen-Lu Chen, Chun-Ju Lin, San-Ni Chen, Ming-Chieh Hsieh, Lei Wan, Peng-Tai Tien

**Affiliations:** 1 Department of Ophthalmology, Eye Center, China Medical University Hospital, China Medical University, Taichung, Taiwan; 2 School of Chinese Medicine, China Medical University, Taichung, Taiwan; 3 School of Medicine, College of Medicine, China Medical University, Taichung, Taiwan; 4 Department of Optometry, Asia University, Taichung, Taiwan

**Keywords:** PDLLA, Vision loss, Filler, Retinal artery occlusion, Hyperbaric oxygen therapy, Case report

## Abstract

**Background:**

Poly-D, L-lactic acid is (PDLLA) a new cosmetic filler. We reported the first case of PDLLA-related devastating complication of multiple branch retinal artery occlusion (BRAO).

**Case presentation:**

A 23-year-old female had sudden blindness after injection of PDLLA at the glabella. After emergency intraocular pressure-lowering medicine, ocular massage, steroid pulse therapy, heparin and alprostadil infusion, and subsequent treatments including acupuncture and 40 sessions of hyperbaric oxygen therapy, her best-corrected visual acuity improved from hand motion at 30 cm to 0.3 within 2 months.

**Conclusion:**

Although safety of PDLLA was evaluated in animal studies and in 16,000 human cases, it could still cause rare but devastating retinal artery occlusion as in the present case. Proper and immediate therapies could still improve patient’s vision and scotoma. Surgeons should keep in mind the possibility of iatrogenic filler-related retinal artery occlusion.

## Background

Injectable poly‐D, L‐lactic acid (PDLLA) is a new cosmetic filler (AestheFill; REGEN Biotech, Inc., Seoul, South Korea). This subdermal stimulatory filler was first approved by the Korean Food and Drug Administration in 2014 and commercially marketed in Taiwan in 2020 [1]. Here, we presented the case of a 23-year-old female, who showed no previous ophthalmic medical history, complicated with sudden vision loss in the right eye after PDLLA injection at the glabella. It is the first reported case of PDLLA-related devastating complication of retinal artery occlusion.

### Case presentation

A 23-year-old postpartum woman was referred to our emergency department (ED) due to right-eye sudden blindness after receiving poly‐D, L‐lactic acid (PDLLA) subdermal filler (AestheFill; REGEN Biotech, Inc., Seoul, South Korea) injection at a clinic. She had a smooth pregnancy period and delivered the baby successfully 2 months ago. She never had blurred vision before. After injection of the PDLLA subdermal filler at the glabella, she had burning sensation on the right forehead away from the injection site, followed by right-eye vision loss within just a few seconds. She also felt dizzy and vomited several times after injection. She was sent to the ED of another medical center and then referred to our hospital.

**Fig. 1:**
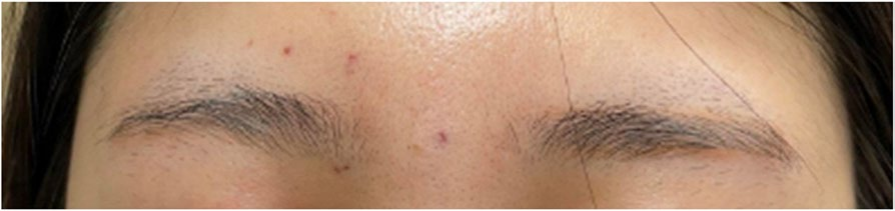
Patient face showed several pinhole wounds without necrosis

During physical examination, her best-corrected visual acuity (BCVA) with Snellen chart was only seeing hand motion at 30 cm of the right eye and 1.0 of the left eye. The patient also had relative afferent pupillary defect of the right eye. There was no corneal edema, ophthalmoplegia, ptosis or any strabismus of the right eye. There were few pinhole scars on the glabella and medial side above and beneath the right eyebrow. No skin necrosis was seen (Fig. 1).

**Fig. 2:**
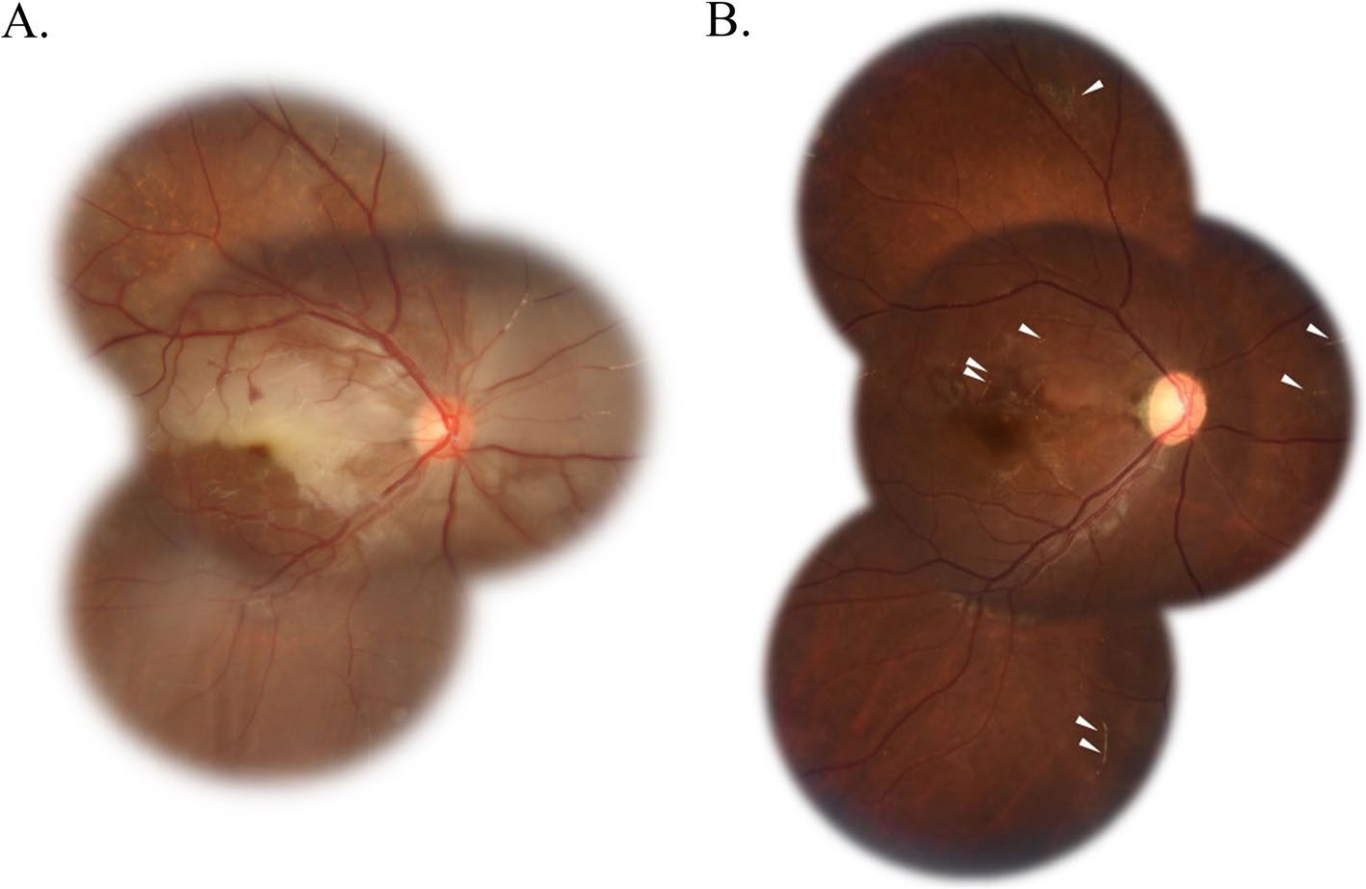
Fundus photographs. **A** retinal artery occlusion multiple filler embolus and sectoral retina whitening. **B** Involved vessels sheathing (arrowheads) and no more retina whitening were noted after two months

The brain and orbital computed tomography scan were performed and the results were within normal limits. The complete blood count, electrolytes, and coagulation profiles were all unremarkable. Fundus photographs showed nasal upper branch retinal artery occlusion and temporal artery occlusion due to multiple filler embolus. Sectoral retinal whitening surrounding the occluded arteries was noted (Fig. 2A).

**Fig. 3:**
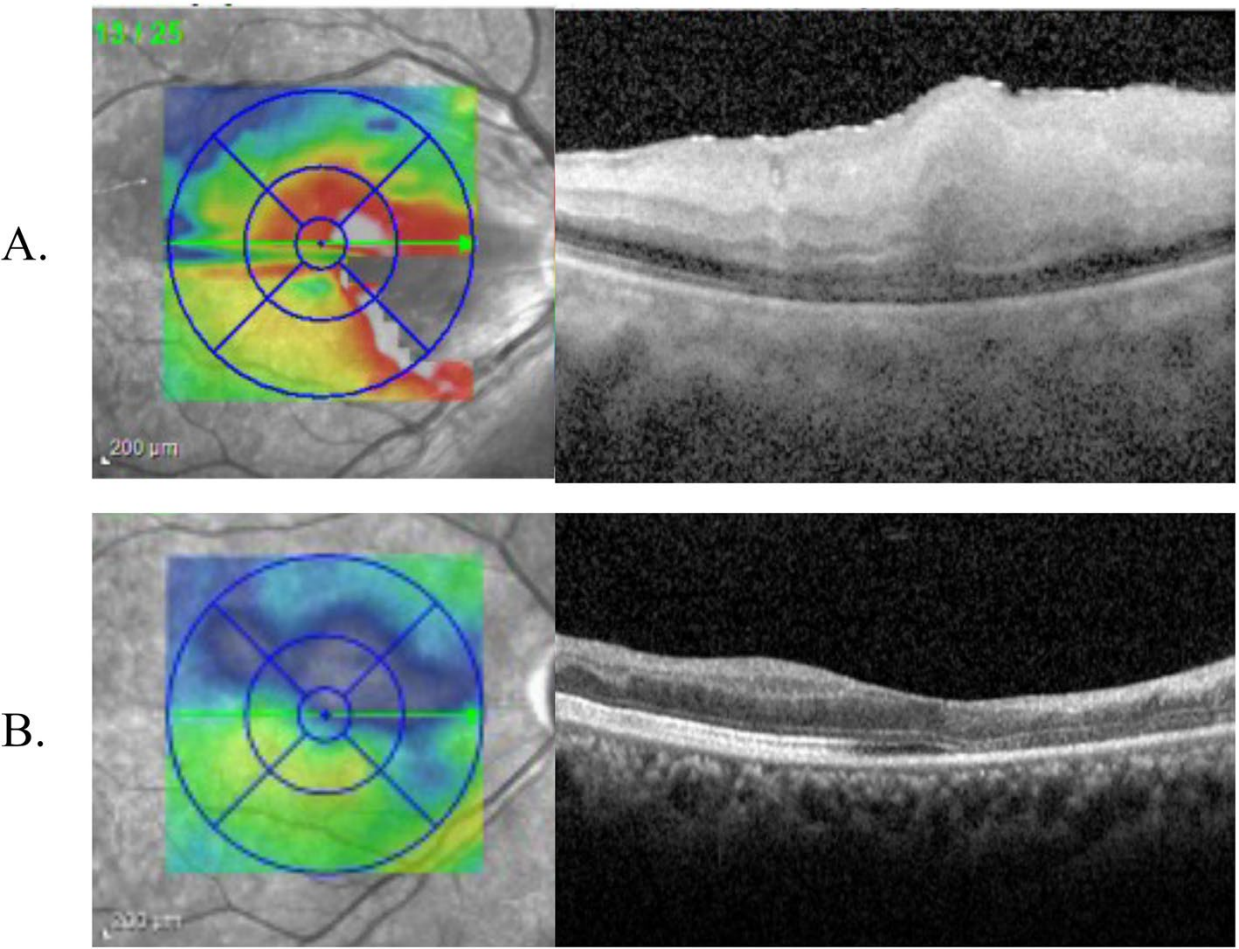
Optical coherence tomography images. **A** Initial images showing typical retinal artery occlusion appearance with inner retina swelling. **B** Two months later, mild ellipsoid zone disruption and inner retina atrophy were noted

**Fig. 4:**
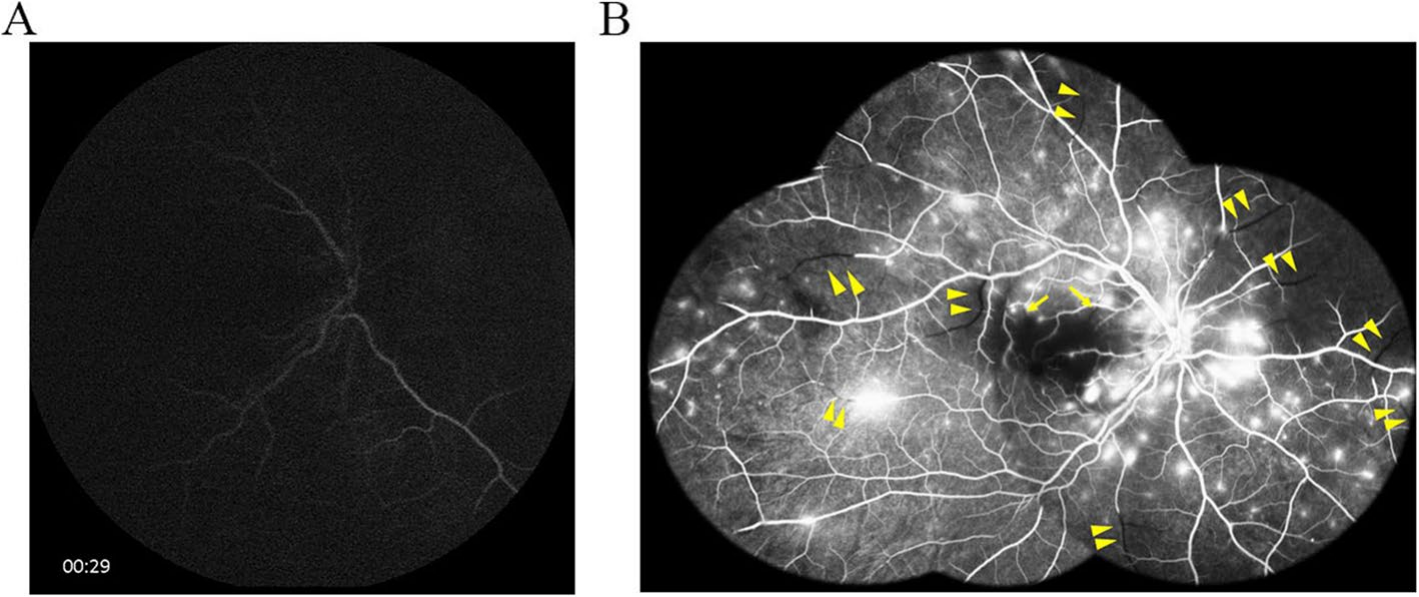
**A** Fluorescein angiography showing no fluorescein entering the artery phase at 29 s; **B** Montage FA picture late-phase showed multiple leakages and occlusion of arteries and arterioles (arrowheads). Notably, the arterioles supplied the central fovea upper part were occluded (arrows) comparable to lower VF defect

Optical coherence tomography images showed increased reflectivity and thickness of the inner retina and a corresponding decrease of reflectivity in the outer layer of the retina and retinal pigment epithelium/choriocapillaris layer (Fig. 3A). Fluorescein angiography (FA) showed delayed vascular filling with late-phase multiple leakages and multiple occluded arterioles with terminal non-perfusion (Fig. 4A, B).

**Fig. 5:**
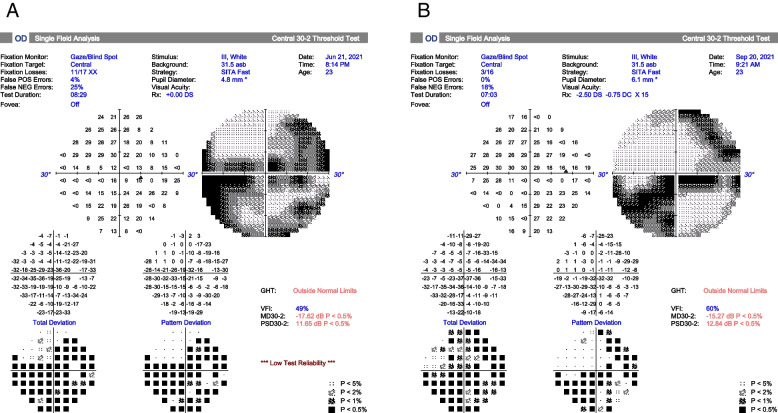
**A** The initial visual field showed multiple scotomas. **B** Less central scotoma after 2 months

At ED, we laid the patient in the supine position and lowered her intraocular pressure using topical Brimonidine tartrate 0.2%/Timolol maleate 0.5% (Combigan, Allergan Inc.) and 5-min ocular massage (repeated pressure applied to the globe for 10–15 s, followed by sudden release). Steroid pulse therapy (methylprednisolone 500 mg four times a day for 3 days) and infusion of heparin and alprostadil were given soon after retinal artery occlusion was confirmed. The next day, her right-eye vision got mild improvement, and her BCVA improved to 0.05. We also arranged hyperbaric oxygen therapy (HBOT) 5 days later, with a total of 40 HBOT sessions administered in the following two months. After two months, her fundus showed no more retina whitening but vessel sheathing and focal atrophy (Fig. 2B). OCT showed inner retina atrophy and ellipsoid zone disruption (Fig. 3B). But surprisingly, her right-eye BCVA improved to 0.3. Improvement of central scotoma was also confirmed by visual field examinations (Fig. 5A, B).

## Discussion and conclusions

Restoring the depleted soft tissue volume using injectable fillers has become increasingly popular nowadays. Polylactic acid (PLA) is an aliphatic polyester. It is widely used for biomedical applications because its degradation products H2O and CO2 are neither toxic nor carcinogenic to the human body [2]. Owing to its safe application and long-lasting effect, PLA substances including poly-L-lactic acid (PLLA) and PDLLA have been rising stars in the cosmetic filler market [3, 4].

Animal studies of PDLLA application were conducted between September 2009 and May 2011. PDLLA microspheres were injected into the subcutaneous space of rats. PDLLA-induced cell inflammation was observed from the 2^nd^ week and subsided in the 4^th^ week. Cells, actin, and type-1 collagen were increasing near and inside the PDLLA microspheres from the 2^nd^ to 20^th^week post-injection. No abnormal finding was found at the injection site. Animal studies proved the efficacy and safety of PDLLA microspheres as subdermal fillers [5]. More studies of PDLLA as a filler were done and showed comparable efficacy and safety with hyaluronic acid for the correction of nasolabial folds [6–8].

Research performed by the manufacturer reported approximately 16,000 patients receiving facial injection of PDLLA filler from April 2014 to July 2018 with no serious adverse effects (i.e., death, blindness, and skin necrosis). Adverse side effects observed included mild swelling (50% of all patients), bruising (30%), and pain (20%) which were resolved or relieved with ice packing and medication [1]. Although previous studies showed safety of PDLLA as a subdermal filler, the present case had a major side effect of the multiple branch arterial occlusion after injection of PDLLA above the eyebrow.

Emergency treatment was administered to the case patient according to suggestions made by the management of a committee convened by the Society of Aesthetic Medicine (Singapore) [9]. They included the immediate lowering of intraocular pressure and dislodging the embolus to a more peripheral downstream location. We laid the patient down and administered one drop of topical Brimonidine tartrate 0.2%/Timolol maleate 0.5%. Followingocular massage that could also lower intraocular pressure, blood flow increased in the arterioles, potentially dislodging the embolus. Loh et al. also recommended supportive treatments including steroid pulse therapy and heparin infusion and HBOTafter immediate treatment [9]. Alprostadil as a vessel dilator had previously been used [10, 11], which helps move the emboli to a more distal part of the retinal artery.

With the outbreak of the coronavirus disease-2019 (COVID-19) pandemic, our HBOT building had been changed into a COVID-19 ward. HBOT was arranged immediately after reopening of the HBOT unit. The meta-analysis study conducted by Wu et al. in 2018 reported beneficial effect of oxygen therapy in improving visual acuity in retinal artery occlusion (RAO) patients, especially those treated with 100% HBOT and for over 9 h [12].

The present case had immediate vision loss after injection at the glabella area. A review study of 98 patients showed glabella (38.8%) as the most common injection site of iatrogenic RAO, followed by nasal region (25.5%), nasolabial fold (13.3%), and forehead (12.2%) [13]. The ophthalmic artery system branches into vessels including the supraorbital, supratrochlear, and dorsal nasal arteries. It has been suggested that injection at the glabella or inferior to the forehead should be superficial while injection on the forehead should be deep because supratrochlear artery and supraorbital artery become more superficial when they travel superiorly above the supraorbital rim [14].

Other prevention tips include aspiration before injection, avoiding overcorrection by using low volumes in two or more treatment sessions, and using botulinum toxin in the glabella first to reduce the severity of wrinkles before injecting soft tissue filler [15, 16].

The pathogenesis of filler-caused RAO can be attributed to intravascular injection or penetrating the proximal branches of the artery or anastomoses vessels. The high pressure of injection can make the filler retrograde to the larger vessel. In our case, the filler retrograded from the supratrochlear artery to the ophthalmic artery then propelled forward to central retina artery and then occluded multiple branch retina arteries [13].

Treatment of RAO within a limited time from onset, known as the golden period, may achieve better VA. In CRAO, experimental monkey model showed the retina can only tolerate up to 97 min of CRA clamping without causing detectable injury [17]. Four hours of arterial occlusion results in massive irreversible retinal injury, therefore, the recanalization therapies were generally suggested within four hours [18]. However, in human, the time window was found to be 15 h in patients with central retinal artery occlusion (CRAO) caused by fibrinoplatelet embolization [15]. Early treatment appears to be as important in BRAO cases as it is in CRAO cases to avoid retinal damage after artery obstruction and ischemia. One study suggests 24 h threshold time for the conventional treatment for BRAO [19]. The present case came to our ED after 5 h of symptom onset and initial visit to another hospital. The ophthalmologist there informed the patient of poor prognosis but performed no treatment. This article aims to bring attention to possible improvement of visual acuity within the human time window.

In conclusion, retinal artery occlusion can be a devastating side effect after cosmetic facial filler injection. Physicians who provide the filler service should pay attention to the location and level of injection, signs and proper referral of iatrogenic RAO. Although safety of PDLLA was confirmed in animal studies and prior application on 16,000 human cases without serious adverse effects, it could still cause the rare but devastating complication of RAO. This is the first reported case of PDLLA-related RAO worldwide. Proper and immediate therapies could still improve patient’s vision and scotoma. Ophthalmologists should keep in mind the possibility of iatrogenic filler-related RAO. Further treatment guidelines and investigations are needed.

## Data Availability

The datasets used and/or analysed during the current study are available from the corresponding author on reasonable request.

## Abbreviations

PDLLA: Poly-D, L-lactic acid
BRAO: Branch retinal artery occlusion
ED: Emergency department
BCVA: Best-corrected visual acuity
HBOT: Hyperbaric oxygen therapy
RAO: Retinal artery occlusion
PLA: Polylactic acid
PLLA: Poly-L-lactic acid
CRA: Central retinal artery
COVID-19: Coronavirus disease-2019
CRAO: Central retinal artery occlusion
